# Irritability Is Associated With Decreased Cortical Surface Area and Anxiety With Decreased Gyrification During Brain Development

**DOI:** 10.3389/fpsyt.2021.744419

**Published:** 2021-09-22

**Authors:** Camille Piguet, Angeline Mihailov, Antoine Grigis, Charles Laidi, Edouard Duchesnay, Josselin Houenou

**Affiliations:** ^1^NeuroSpin, CEA, University Paris Saclay, Gif-sur-Yvette, France; ^2^Department of Psychiatry, Faculty of Medicine, University of Geneva, Geneva, Switzerland; ^3^Université Paris Est Créteil, INSERM, IMRB, Translational Neuropsychiatry, Fondation FondaMental, Créteil, France; ^4^Assistance Publique-Hôpitaux de Paris (AP-HP), DMU IMPACT, Mondor University Hospitals, Créteil, France

**Keywords:** irritability, anxiety, MRI, development, gyrification index, dimensional model

## Abstract

**Background:** Brain development is of utmost importance for the emergence of psychiatric disorders, as the most severe of them arise before 25 years old. However, little is known regarding how early transdiagnostic symptoms, in a dimensional framework, are associated with cortical development. Anxiety and irritability are central vulnerability traits for subsequent mood and anxiety disorders. In this study, we investigate how these dimensions are related to structural changes in the brain to understand how they may increase the transition risk to full-blown disorders.

**Methods:** We used the opportunity of an open access developmental cohort, the Healthy Brain Network, to investigate associations between cortical surface markers and irritability and anxiety scores as measured by parents and self-reports.

**Results:** We found that in 658 young people (with a mean age of 11.6) the parental report of irritability is associated with decreased surface area in the bilateral rostral prefrontal cortex and the precuneus. Furthermore, parental reports of anxiety were associated with decreased local gyrification index in the anterior cingulate cortex and dorsomedial prefrontal cortex.

**Conclusions:** These results are consistent with current models of emotion regulation network maturation, showing decreased surface area or gyrification index in regions associated with impaired affective control in mood and anxiety disorders. Our results highlight how dimensional traits may increase vulnerability for these disorders.

## Introduction

The emergence of severe psychiatric disorders, in particular during adolescence, has been the focus of a rising interest in psychiatric neuroscience research (1). With a better understanding of psychiatric diseases owing to knowledge from neuroimaging studies in particular, new effort is put toward deciphering the apparition of prodromal symptoms and defining useful clinical staging models (2). However, it is clear now that no single disease pathway can be found, and that common prodromal symptoms may crystallize into different phenotypes (3), depending on complex genetic, environmental, and epigenetic factors. Rather, different signs and symptoms can be regarded as common precursors, emphasizing the need to study them as vulnerability factors. In this context, the dimensional model (RDoC) has gained significant popularity. It postulates the isolation of relevant clinical symptoms, or prodromal signs, with increased probability to relate them to biological underpinnings. This is central for early detection and diagnosis, leading to improved treatment and outcome (3).

*Irritability*, as defined by increased proneness to anger relative to peers (4), and *anxiety* are excellent candidates in this respect (5). Both have been associated with emotion dysregulation and high levels of psychopathology (6, 7). However, they seem to project on different latent variables in the context of developmental emotion regulation disorders (8, 9). Irritability is a clinical predictor of both internalizing and externalizing disorders, and a symptom of numerous disorders in child and adolescent psychiatry (10). Associated in particular with Disruptive Mood Dysregulation Disorder (DMDD) when extremely pathological, but also with Opposition Defiant Disorder (ODD) in children, and importantly with depression and anxiety later in life, irritability predicts an elevated risk for negative outcomes and functional impairment in adulthood (11). Nevertheless, its relationship with mood dysregulation, externalized behavior and suicidality makes it a central dimension for which a common pathophysiological mechanism is supported by translational models (6).

Elevated levels of trait anxiety have been pointed out as a prodromal symptom in bipolar disorder (12), schizophrenia (13), and both trait anxiety and anxiety disorders predict depression later in life (14, 15). If anxiety symptoms generally decrease between early and middle adolescence, subgroups of adolescents that present continuous anxiety symptoms are more at risk to develop anxiety disorders (16). Even subthreshold anxiety symptoms are associated with higher levels of psychopathology and suicide risk (17). Higher trait anxiety is therefore a non-specific but central marker of vulnerability in mental health disorders.

Based on these findings, it is therefore expected that relevant brain abnormalities would be associated with prodromal traits of irritability, on the one hand, and anxiety, on the other hand, in children and adolescents. However, neuroimaging studies so far are still sparse with conflicting results (8, 9, 18–20). Though, brain signatures of irritability in a large population-based developmental cohort still remained to be identified.

Regarding structural correlates of trait anxiety, findings in adolescents have been so far limited and inconsistent, mainly focusing on the ventromedial PFC (vmPFC) (21). Some studies have correlated behavioral inhibition, a proxy for anxious temperament, and brain structure. For example, a longitudinal study found that behavioral inhibition during early childhood predicted lower cortical thickness in the dorsal ACC/dmPFC in young adults, though independently from the anxiety measure (22). Anxiety severity during adolescence was associated with a thicker ventrolateral prefrontal cortex (vlPFC). However, another study found larger vlPFC volume to be associated with less severe symptoms of social anxiety (23). With anxiety representing a multifaceted concept, and limited studies in youth, no general conclusion can be drawn so far, emphasizing the need to test different markers of structural brain maturation.

Besides cortical thickness, other indices of brain maturation may have their importance when studying brain development. Complementary to cortical thickness, surface area and gyrification indices have proven to be associated with different genetic and environmental variables (24). The local gyrification index might be more representative of early brain development, and hence represent a good index of early vulnerability (25). Together, these metrics allow for a more precise quantification of neurodevelopment than gray matter volume (26) and remained to be used in the context of irritability and anxiety.

## Materials and Methods

### Participants

The HBN cohort started in 2015, with the aim of recruiting 10,000 children aged 5–21 from the community, using advertisements to encourage the participation of children whose parents have concerns about their school results or potential psychiatric problems (27). Written informed consent was obtained from participants aged 18 or older, and from legal guardians, in addition to themselves, for those under 18 years old. This protocol was approved by the Chesapeake Institutional Review Board, is conducted following the Declaration of Helsinki for human research is described in the following reference: https://doi.org/10.1038/sdata.2017.181.

Main exclusion criteria were IQ <66 and severe developmental problem or handicap, recent (last 6 months) psychotic disorder (schizophrenia, bipolar, schizoaffective), or new onset suicidality without on-going treatment. IQ was measured using the Wechsler Adult Intelligence Scale (WAIS-III) or the Wechsler Intelligence Scale for Children (WISC-III) depending on age. During the course of the acquisition phase of the study, new data was continuously released based on a rich phenotyping protocol. We downloaded the neuroimaging and clinical data from the study portal (http://fcon_1000.projects.nitrc.org/indi/cmi_healthy_brain_network/sharing_neuro.html) from the first three releases, including a total number of 1,552 subjects, in October 2018.

### Neuroimaging

Participants completed a T1 MRI as part of the HBN protocol (http://fcon_1000.projects.nitrc.org/indi/cmi_healthy_brain_network/mri_protocol.html). Details of the image acquisition and processing protocols are provided on the HBN portal, and included a standardized high-resolution structural MRI protocol on 3 sites in NYC area: a mobile 1.5T Siemens Avanto in Staten Island, a 3T Siemens Tim Trio at Rutgers University Brain Imaging Center, and a 3T Siemens Prisma at the CitiGroup Cornell Brain Imaging Center. Following preprocessing and segmentation procedures using Freesurfer software version 6.0.0 (http://surfer.nmr.mgh.harvard.edu), quality control procedures were applied. A quality check was conducted on our neuroimaging data both manually via visual inspection of each segmented scan, as well as using FreeSurfer's Euler number, which measures the topological complexity of our reconstructed cortical surfaces (28). Since neuroimaging data on children are often more difficult to acquire, many subjects showed significant movement artifacts. Nevertheless, the correlation between manual inspection and automated Euler number's cut-off showed a very good correlation. Data not passing quality checks were deemed unacceptable for inclusion, resulting in available data from 860 participants. In order to gain in homogeneity, we also retained only subjects with IQ >70. The intersection between these subjects and good quality imaging data yielded a sample of 718 subjects participating in our neuroimaging analyses. Subsequently, 4 subjects that had a good segmentation data failed the preprocessing step, because of corrupted associated files. Furthermore, after selecting scores of interest, we finally obtained 645 subjects with available ARI-parents data, 658 with ARI-self data, 594 with available SCARED-parents data, and 527 with SCARED-self data.

Using FreeSurfer, cortical thickness (CT) was calculated as the shortest distance between the white and pial surfaces. Surface area (SA) is calculated as the sum of the area of all triangles surrounding the vertex. The local gyrification index (LGI) is measured as the ratio between buried and visible cortex (29). We used all three indices of cerebral maturation since they have different developmental trajectories (30).

### Statistical Analysis

Given the wide variability in the results reported so far in the literature, we decided to use a whole brain approach. Vertex-wise statistical analyses were conducted using the command-line group analysis stream in FreeSurfer. Cortical surfaces for each participant were first registered to the freesurfer *fsaverage* template, then smoothed using a full-width-at-half maximum (FWHM) kernel of 10 mm for cortical thickness and surface area, and 5 for the local gyrification index.

**Figure 1:**
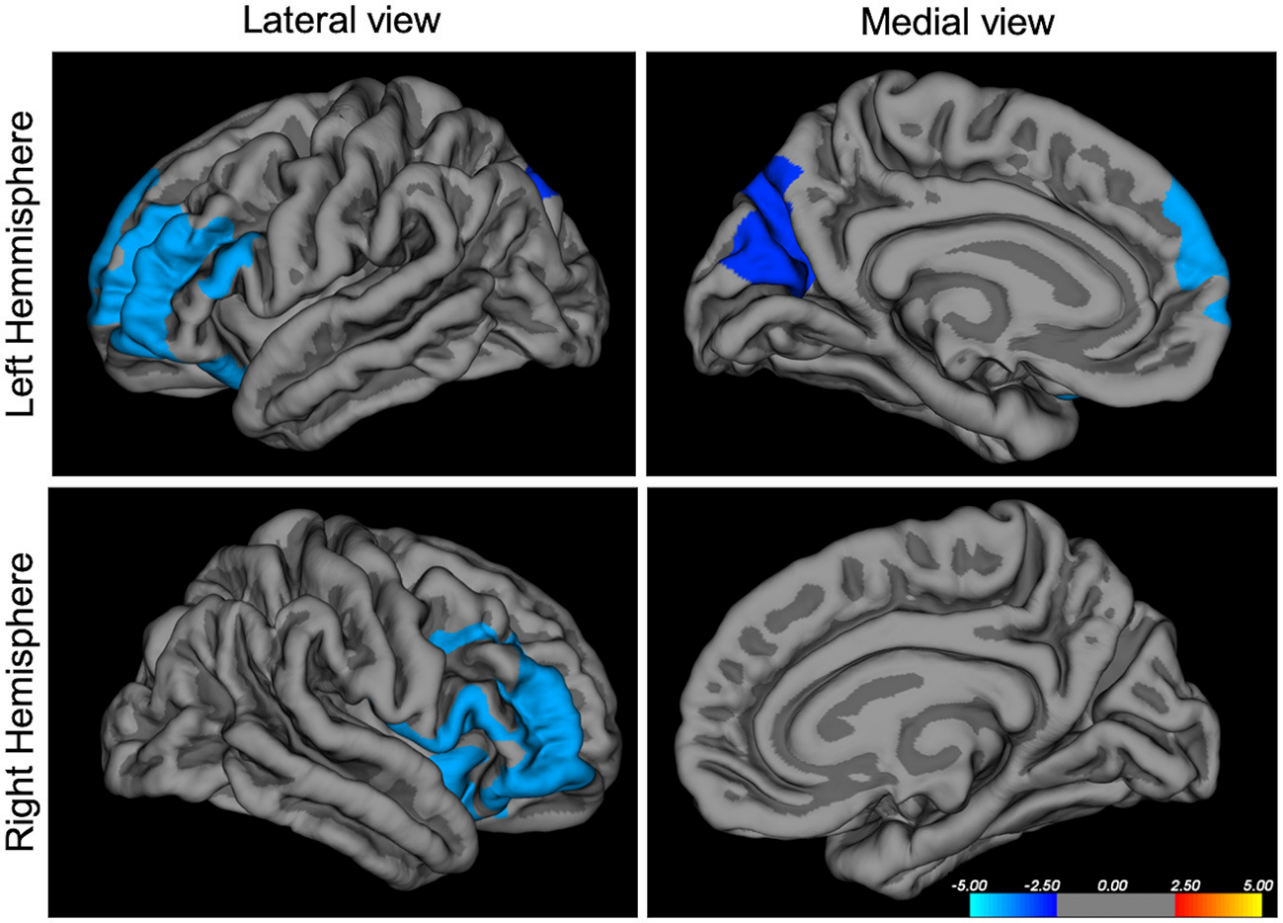
Negative correlation between ARI-parents score and cortical surface area in mm^3^, *p* < 0.05 corrected, *N* = 641. ARI, Affective Reactivity Index. The colors represent the –log^10^ (*p*-value at cluster level) with red indicating a positive correlation and blue indicating a negative correlation between scores and morphological features.

**Figure 2:**
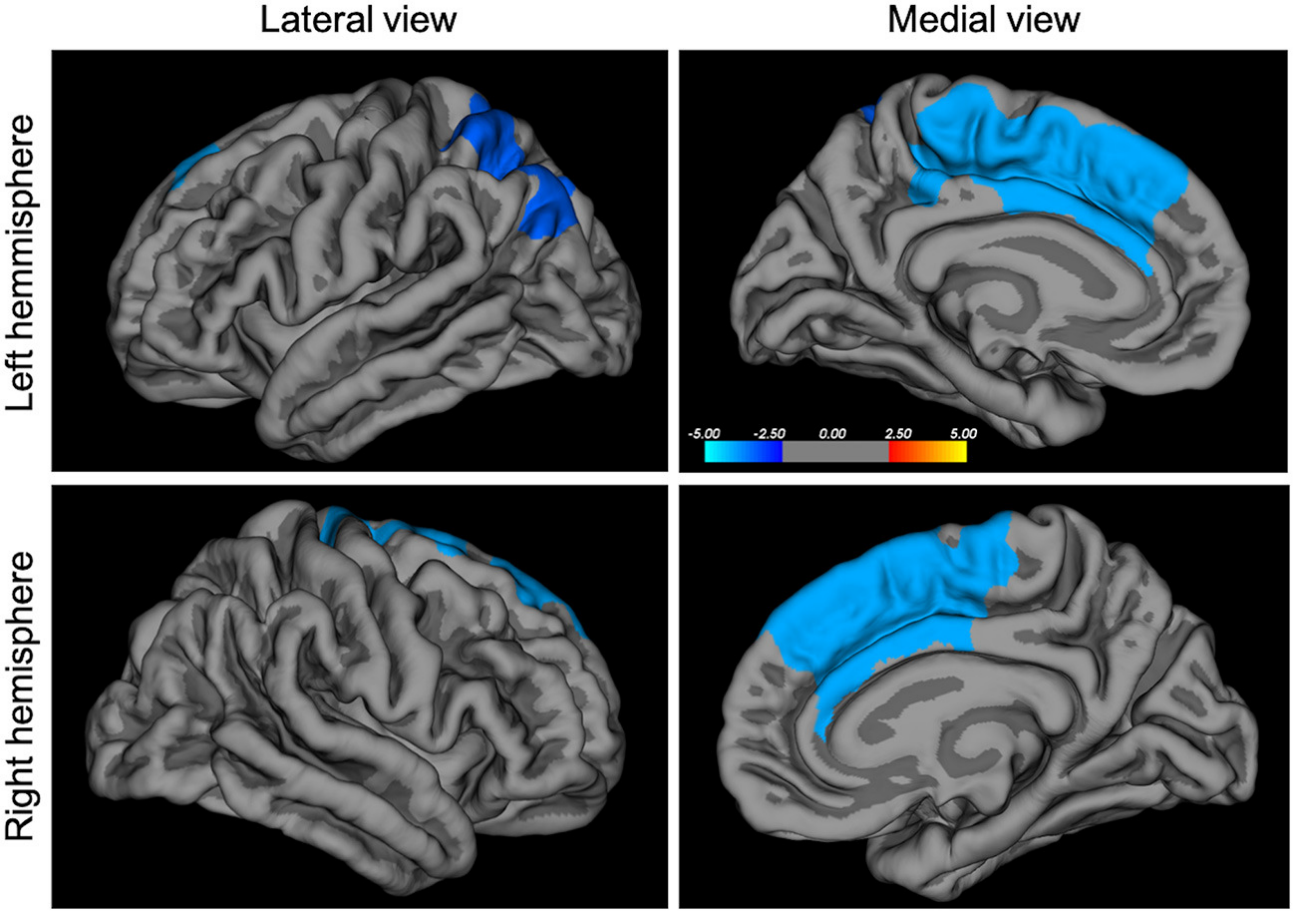
Negative correlation between SCARED-parents score and local gyrification index, *p* < 0.05 corrected, *N* = 590. SCARED, Screen for Child Anxiety Related Emotional Disorders. The colors represent the –log^10^ (*p*-value at cluster level) with red indicating a positive correlation and blue indicating a negative correlation between scores and morphological features.

A general linear model was fit at each vertex to correlate each cortical feature (cortical thickness, surface area, gyrification) with the clinical score of interest, taking into account gender and MRI acquisition site as categorical covariates, and age and IQ as continuous covariates. Correction for multiple comparison at a cluster-wise *p*-value of *p* < 0.05 were conducted using a Montecarlo simulation (10,000 iterations), using the mri_glmfit-sim precomputed at a minimum cluster-wise forming threshold of *p* = 0.01. Therefore, we used two steps of corrections for multiple comparisons, first the *cluster-forming threshold* that calculates significance based on *p*-value of clusters rather than the value of independent vertices, then we ran a *cluster-wise threshold*, removing clusters that are most likely present due to chance. For the sake of clarity, we present our results in Figures 1, 2 with colors representing the –log^10^ (*p*-value at cluster level). Therefore, the values are constant within each particular cluster but they may change between different clusters.

### Questionnaires

The Affective Reactivity Index (ARI) questionnaire was filled by both the participant and their parents (31). ARI is a seven-item questionnaire assessing irritability during the last 6 months (six items measure symptoms and one measures functional consequence). Responses are scored on a three-point scale, from 0 (not true) to 2 (certainly true), and the 6 symptomatic items are summed to create a total score (max = 12 points).

The Screen for Child Anxiety Related Emotional Disorders (SCARED) questionnaire (32) was also filled by participants and their parents. It consists of 41 items rated on a three-point scale, and questions various aspects of anxiety over the past 3 months (a score of 25 and above can indicate an anxiety disorder).

## Results

### Demographic

Mean age is around 11–12 years old, depending on the subsample for ARI parents report, ARI self-report, SCARED parents report or SCARED self-report (Table 1). The proportion of males is almost 60%.

**Table 1:** Demographic repartition of each of the subgroups from separate analyses; mean scores of questionnaires and standard deviation; ARI, Affective Reactivity Index; SCARED, Child Anxiety Related Emotional Disorders.

**Score type**	***N***	**Mean age (SD)**	**% male (*SD*)**	**Mean score (*SD*)**	**IQ (*SD*)**
ARI_P	645	11.3 (3.4)	59.4	3.2 (3.4)	100.16 (15.8)
ARI_S	658	11.4 (3.6)	58.8	3.6 (3.3)	100.25 (15.7)
SCARED_P	594	11.6 (3.4)	59.0	15.2 (12.1)	98.34 (16.6)
SCARED_S	527	12.2 (2.9)	58.1	23.2 (15.6)	98.07 (16.4)

*_P, parents report; _S, self-report; N, Subjects number; SD, standard deviation; IQ, intelligence quotient*.

### Questionnaires

The ARI scores exhibited a high correlation [*r*_(645)_ = 0.31, *p* < 0.001] between parents and self-reports. Mean score for self-report was 3.6 (*sd* 3.3) and mean score for parents reports was 3.2 (*sd* 3.4), see Table 1. SCARED scores also correlated significantly between parents and children reports [*r*_(527)_ = 0.24, *p* < 0.001]. A correlation was found between the ARI and SCARED self-reports [*r*_(527)_ = 0.34, p <0.001], and between ARI and SCARED parents reports [*r*_(594)_ = 0.3, *p* < 0.001] (see Supplementary Material for distributions and correlations of the questionnaires).

### Neuroimaging

A significant negative correlation was observed between the ARI parents' reported scores and surface area in the bilateral rostrolateral prefrontal cortex (inferior and middle frontal gyrus, Brodmann area 10), and in the precuneus (Figure 1 and Table 2), surviving a statistical cluster-forming threshold of *p* < 0.05, corrected. ARI self-report scores negatively correlated with inferior occipital gyrus thickness at *p* < 0.05, however these results did not survive corrections for multiple comparisons.

**Table 2:** Main results; ARI-P, Affective Reactivity Index, Parents' report; SCARED-P, Screen for Child Anxiety Related Emotional Disorders, Parent's report; MNI, Montreal Neuroimaging Institute; LGI, Local Gyrification Index.

	**MNI (x, y, z)**	**Peak region**	**Size (mm^**2**^)**	**Features**
**ARI-P**				
Left	−33, 50, 11	rostralPFC	5,930	↓surface area
	−11, −74, 46	precuneus	2,543	↓surface area
Right	40, 50, −8	rostralPFC	5,726	↓surface area
**SCARED-P**				
Left	−9, 12, 33	mid ACC/dmPFC	4,910	↓LGI
	−34, −77, 43	inf. parietal gyrus	3,245	↓LGI
Right	11, 16, 40	mid ACC/dmPFC	4,837	↓LGI

*↓decreased*.

We report a significant negative correlation between SCARED parents' report and the local gyrification index in the dorsomedial prefrontal gyrus/middle cingulate gyrus and in the inferior parietal gyrus, at *p* < 0.05, corrected (Figure 2 and Table 2). No significant correlation was found for the SCARED self-report measure.

## Discussion

In this study, we used the open access data from a population-based sample of children and adolescents (enriched for psychopathology) to assess structural correlates of irritability and anxiety, which are important transdiagnostic vulnerability traits for a wide range of psychiatric disorders. In a sample of 658 subjects, we found a strong correlation between irritability, as reported by the parents, and decreased surface area in the rostral prefrontal cortex bilaterally, and a weaker correlation with decreased surface area in the precuneus. We also found a strong negative correlation between parents' report of anxiety and cortical gyrification in the rostral ACC/dorsomedial PFC. These regions are very important for emotion regulation, a process strongly dependent on brain development. Interestingly, we were able to show that irritability and anxiety do not display similar structural correlates, supporting their independence as separate dimensions (8, 9). Self-reports yielded no association that survived corrections for multiple comparisons, despite being behaviorally correlated with parent's reports. However, the literature supports the fact that parents' and children's (and teachers') reports present only low to moderate correlations (33). Recent studies have also used the parents' report (19), or an average of both parents' and children's reports (34). Using the same scales as measures of irritability and anxiety, a study on a transdiagnostic cohort showed that self-report and parent's report are projected on different latent variables (9). They concluded that self-reports might be underestimated, and showed no structural brain correlation in these 197 youths either. One might hypothesize that parents' reports better represent true psychopathological symptoms since it has been shown that they tend to correlate more strongly with diagnosis information (35). Importantly, our results show that these two different dimensions are associated with separate parts of the emotion regulation network.

The study of neural underpinnings of irritability in youth has produced heterogeneous results so far, probably given the differences in samples and methods. However, the prefrontal cortex has repeatedly been associated with irritability, such as seen in one of the first studies targeting irritability, which analyzed Voxel-Based Morphometry in 78 Severe Mood Dysregulation (SMD) children, 55 youth with BD, and 68 healthy controls (36). In both clinical groups, authors found a decrease in gray matter volume in the pre-supplementary motor area, the insula and the dlPFC, with additional changes in other brain regions for the BD group. On the other hand, a study from the IMAGEN consortium found a correlation between irritability and decreased gray matter volumes in the rostral inferior frontal gyrus in 14-year-old adolescents (20). A study with a similar design ran a multivariate analysis of non-negative matrix factorization and found decreases in cortical thickness in the orbitofrontal cortex and temporal lobe regions (18). However, these results cannot be directly compared to the results presented here, obtained with a classical and conservative Freesurfer analysis. Interestingly, in the study of Jirsaraie et al., effects in the dorsolateral cortex seemed more pronounced in the younger subsample of the cohort, which is in line with results obtained in our study, showing a strong bias toward younger preadolescents, with a mean age of 11.6 years old. Additionally, a study looking at dimensional ADHD symptoms using a multimodal imaging approach reported that one component independent from ADHD symptoms was irritability, as measured by the ARI questionnaire, and was associated with decreased volume in superior frontal areas (37). Therefore, with some variations, irritability has repeatedly been associated with impairments in rostral frontal cortices, regions often associated with cognitive and affective control ability in the functional neuroimaging literature. More precisely, activity in the lateral frontal poles, as found here, have been associated with emotional action control (38). This is consistent with the literature in disorders presenting with emotion dysregulation such as Bipolar Disorder (BD) and Borderline Personality Disorder (BPD). For example, diminished activity in the ventrolateral prefrontal cortex is considered a hallmark of emotion dysregulation in BD (39), and advocated as better emotion regulation capacities. Although the relationship between structure and function cannot be reduced to a linear relationship between surface area and function, these types of results suggest that findings of decreased surface area in this particular region might be related to impaired cognitive control over emotions and therefore irritability. This is also in line with findings in borderline personality literature, a disorder where two of the main symptoms are affective lability and impulsivity. A multimodal meta-analysis revealed both functional and structural impairments in the rostral prefrontal cortex of these patients (40). Interestingly, a study evaluating responses to Dialectical Based Therapy in borderline personality disorder patients showed a decrease in activity in the rostral prefrontal cortex during inhibitory control (Go No-Go task) in 29 patients (41). This particular region also predicted treatment completion and response, and is thought to be associated with affective control. Smaller volume in the rostral medial PFC has also been associated with conduct disorders, independently of an ADHD diagnosis, in two different meta-analyses (42, 43), again underlying the potential association between decreased volume in this region and impaired cognitive control. Interestingly, one meta-analysis also reports diminished volume in the left precuneus for these patients, in line with our findings of diminished surface area in the precuneus (42). This region is involved in many processes, including affective relevance of a stimulus and in self-processing. It has been previously associated with irritability in an age-dependent manner, showing that activity in the precuneus was increased with irritability in younger adolescents (34). Therefore, the precise role of the precuneus and its shaping across development remains to be explored.

Our reports of diminished surface area in the rostral prefrontal cortex and precuneus in children and adolescents with higher levels of irritability may represent a marker of problematic development of affective control, which represents the cognitive control needed over emotion processing to develop proper emotion regulation abilities. If true, this marker should be more specifically present in prodromal phases of emotion dysregulation disorders thus warranting further research.

The other major finding reported in this study is a strong association between higher levels of anxiety and decreased gyrification in the dorsomedial prefrontal cortex, including part of the anterior cingulate gyrus. As stated before, the local gyrification index might be a marker of early developmental processes (26), although it might also reflect other factors, the respective influence of various types of stressors being not completely understood (44). Findings of decreased gyrification has been linked to measures of cortical connectivity, both functional and structural (45) within the tension-based theory of gyrification (46). However, alternative models explaining cortical gyrification are emerging (47). Interestingly, in a similar cohort of high-risk youth between 9 and 25 years old, in those having a parent with a severe psychiatric disorder, a diminished gyrification index across all brain areas was associated with psychotic symptoms (48). In another study in young adults, trait anxiety was associated with decreased gyrification in the left parietal gyrus, a result that we report as well (49). Since local gyrification index is asymmetrical during early postnatal ages in humans (50), this raises the question of functional and structural asymmetry of the cortex in relation with specific symptoms, warranting further research.

Both ACC and dmPFC have been regularly associated with emotion regulation. In one study, trait anxiety correlated positively in healthy subjects with activity in the dorsomedial PFC during a stress task (51). On the other hand, a meta-analysis of cognitive reappraisal showed that anxiety disorder patients presented robust difficulties in the recruitment of the dorsomedial PFC, together with ACC and the parietal cortex, compared to healthy controls (52). The dorsomedial PFC does not appear to show many age-related local gyrification index alterations (53), which is in line with our results. Interestingly, a meta-analysis of VBM results for adult patients presenting Generalized Anxiety Disorder (GAD) showed decreased volume in ACC, but increased volume in the inferior parietal gyrus among other results, whereas the task-related meta-analysis showed consistently increased activity in the amygdala, and decreased activity in rostral PFC (54). This reflects the degree of heterogeneity still observed in anxiety research, a fact that could be in line with the numerous comorbidities and treatments varying from one study to another. This supports the necessity to investigate anxiety in younger cohorts. Despite conflicting results in adult patients, our results show that the dimension of anxiety as reported by the parents in a developmental cohort represents a unique vulnerability marker. Indeed our results support the model of negative bias, one hallmark of anxiety, being the results of a complex interaction between dmPFC/ACC and amygdala (55). In this model, inhibitory connectivity between dmPFC/ACC and amygdala develops during adolescence and is necessary to downregulate negative bias. If this process is impaired, it could explain the emergence of anxiety disorders during adolescence. Interestingly, based on functional literature, our results also fit with the model suggested by L. Williams, based on RDoC dimensions for anxiety and mood disorders. In this model, one dimension is impaired cognitive control, supported by diminished prefrontal/anterior frontal activity as part of a larger network, and another dimension is threat dysregulation, supported by diminished ACC/dmPFC activity in the insula and amygdala (56). We believe that our results support these mechanistic models, therefore next steps would involve conducting connectivity analyses in the present high-risk cohort in order to further test these suggested brain—behavior associations.

## Limitations

Because of its cross-sectional and correlational design, this study does not allow us to draw conclusions on the causality between brain changes and the clinical dimensions observed. The large age range, including puberty, is another limitation. Age was, however, taken into account in our analyses and we are confident that the strong correlations found are representative of a trait vulnerability. The data from the HBN cohort are open-access and our results could therefore be easily replicated. Given the complications of overlapping symptom dimensions, especially during development, the research domain criteria (RDoC) framework emphasizes the need for dimensional research. Therefore, though we chose to focus on the neural circuitry of transdiagnostic symptoms such as irritability and anxiety, this choice does not fully reflect the complexity of developmental psychopathology.

## Conclusion

In a heterogeneous cohort of children and adolescents enriched for psychopathology, we found a strong correlation between important clinical dimensions of vulnerability and markers of brain development. Irritability levels as reported by the parents of affected children were associated with decreased volume in the rostral frontal cortex, which might explain the diminished cognitive control seen in irritability and impulse-control disorders. Anxiety, on the other hand, as reported by parents, was associated with a decrease in gyrification in the dorsomedial PFC, a region functionally associated with anxiety disorders. Therefore, these results validate these two dimensions as markers of early vulnerability in association with impaired brain development in regions involved in cognitive control. These specific circuits and related behavioral traits should therefore be studied in the development of more targeted and effective early interventions in the domain of emotion dysregulation disorders.

## Data Availability Statement

The datasets presented in this study can be found in online repositories. The names of the repository/repositories and accession number(s) can be found at: http://fcon_1000.projects.nitrc.org/indi/cmi_healthy_brain_network/index.html.

## Ethics Statement

This study involving human participants was reviewed and approved by Chesapeake Institutional Review Board. Written informed consent to participate in this study was provided by the participants' legal guardian/next of kin.

## Author Contributions

Experimental design and statistical analyses were conducted by CP, AM, and ED. Neuroimaging data were processed by AG and AM. Quality control of the structural neuroimaging data was done by AM and CP. This manuscript was prepared by CP, AM, CL, and JH. All authors contributed to the interpretation of the results, as well as to the editing and approval of the manuscript content.

## Funding

This work was supported by the Fondation Fondamental Suisse, Geneva, Switzerland.

## Conflict of Interest

The authors declare that the research was conducted in the absence of any commercial or financial relationships that could be construed as a potential conflict of interest.

## Publisher's Note

All claims expressed in this article are solely those of the authors and do not necessarily represent those of their affiliated organizations, or those of the publisher, the editors and the reviewers. Any product that may be evaluated in this article, or claim that may be made by its manufacturer, is not guaranteed or endorsed by the publisher.
