# Supplementary material for: Irritability Is Associated With Decreased Cortical Surface Area and Anxiety With Decreased Gyrification During Brain Development
Source: Front Psychiatry. 2021 Sep 22;12:744419. doi: 10.3389/fpsyt.2021.744419 (PMC8492928; doi:10.3389/fpsyt.2021.744419)

Supplementary data for "Irritability is associated with decreased cortical surface area and anxiety with decreased gyrification during brain development"

Piguet C, Mihailov A, Grigis A, Laidi C, Duchesnay E, Houenou J

1. Distribution and correlation between self-reported measures; ARI : Affective Reactivity Index ; SCARED: Child Anxiety Related Emotional Disorders

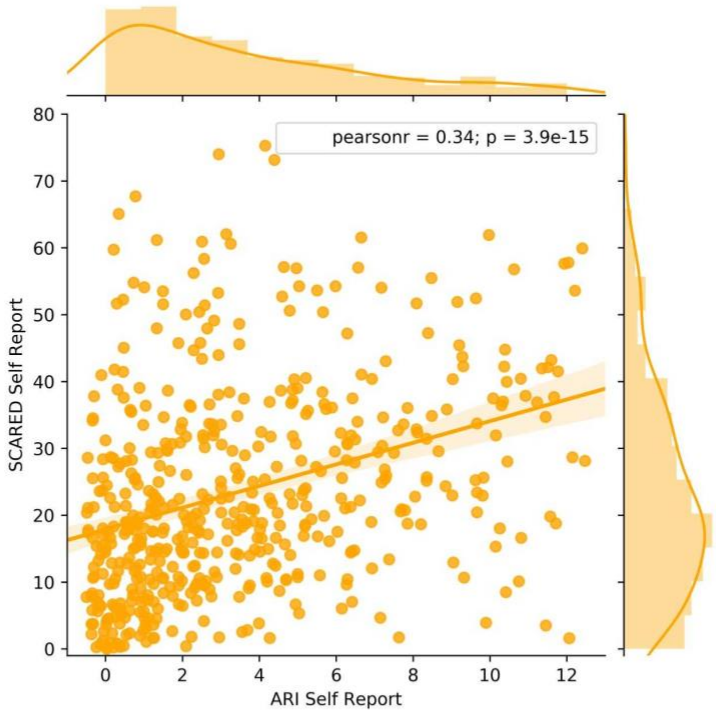

2. Distribution and correlation between parents-reported measures; ARI : Affective Reactivity Index ; SCARED: Screen for Child Anxiety Related Emotional Disorders

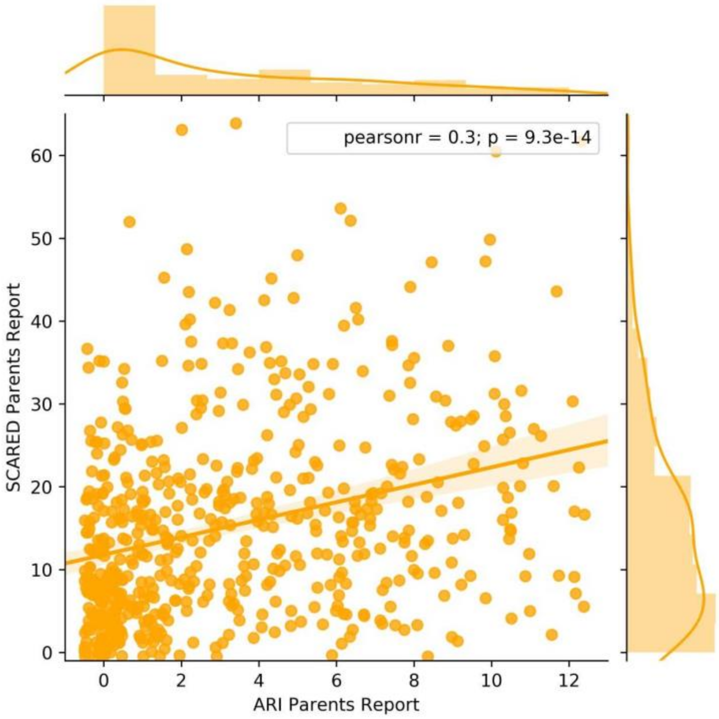

3. Irritability: correlation between self and parents' reports; ARI : Affective Reactivity Index

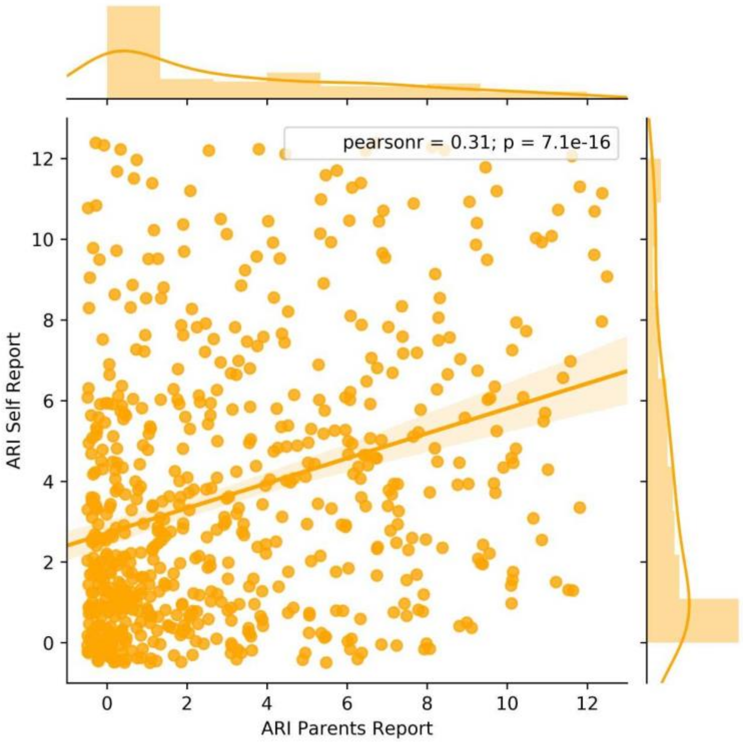

4. Anxiety : correlation between self and parents' reports; SCARED: Screen for Child Anxiety Related Emotional Disorders

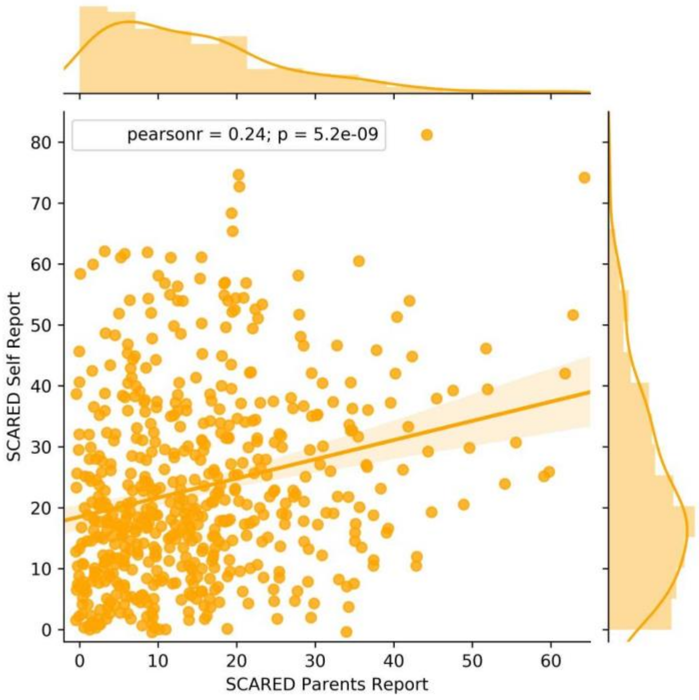

Supplement: Supplementary file 1 [file Data_Sheet_1.pdf]
